# Targeting Myocardial Fibrosis—A Magic Pill in Cardiovascular Medicine?

**DOI:** 10.3390/pharmaceutics14081599

**Published:** 2022-07-30

**Authors:** Alina Scridon, Alkora Ioana Balan

**Affiliations:** 1 Physiology Department, University of Medicine, Pharmacy, Science and Technology “George Emil Palade” of Târgu Mureș, 540139 Targu Mures, Romania; alkora.balan@gmail.com; 2 Emergency Institute for Cardiovascular Diseases and Transplantation of Târgu Mureș, 540136 Targu Mures, Romania

**Keywords:** antifibrotic strategies, cardiac fibrosis, cardiovascular diseases, fibrosis pathways, therapeutic strategies

## Abstract

Fibrosis, characterized by an excessive accumulation of extracellular matrix, has long been seen as an adaptive process that contributes to tissue healing and regeneration. More recently, however, cardiac fibrosis has been shown to be a central element in many cardiovascular diseases (CVDs), contributing to the alteration of cardiac electrical and mechanical functions in a wide range of clinical settings. This paper aims to provide a comprehensive review of cardiac fibrosis, with a focus on the main pathophysiological pathways involved in its onset and progression, its role in various cardiovascular conditions, and on the potential of currently available and emerging therapeutic strategies to counteract the development and/or progression of fibrosis in CVDs. We also emphasize a number of questions that remain to be answered, and we identify hotspots for future research.

## 1. Introduction

Cardiovascular diseases (CVDs) are the leading cause of death and morbidity, accounting for up to one-third of deaths worldwide. The prevalence of CVDs has seen a tremendous increase over the past decades, with a doubling of CVD cases between 1990 and 2019 [1]. In parallel, cardiovascular mortality has also gradually increased during this period, from 12.1 million in 1990 to 18.6 million in 2019 [1]. Metabolic, behavioral, environmental, and social factors have all been linked to increased cardiovascular risk. Whereas several of those factors are modifiable and their removal may lower the prevalence of CVDs, others, such as age, race, sex, or family history, continue to have a significant impact on the evolution of CVDs’ prevalence [1].

Initially seen as an adaptive process designed to ensure wound healing and tissue repair following injury, myocardial fibrosis is now recognized as a major contributor to CVDs and CVD-related morbidity and mortality in many clinical settings [2]. Accumulating data show that most CVDs involve pathological myocardial remodeling characterized by cardiac fibrosis. In myocardial infarction, fibrosis develops as a repair mechanism for maintaining the integrity of the cardiac wall. However, over the long term, the lack of contractile capacity of the fibrous tissue along with the death of cardiac myocytes eventually lead to impaired cardiac function [2]. In many other CVDs (e.g., hypertensive heart disease, diabetic, dilated, and hypertrophic cardiomyopathy, heart failure, chronic ischemic heart disease, or cardiac arrhythmias), fibrosis is also recognized at present as a causative or at least as an aggravating factor [2]. In addition, the natural process of aging promotes cardiac fibrosis via countless pathophysiological pathways, even in the absence of concomitant heart disease [2]. Myocardial fibrosis has thus rose as a promising diagnostic and prognostic marker in CVD patients, and strategies aiming to prevent, halt, or even reverse fibrosis have emerged as promising means to prevent and/or treat various forms of CVD.

This paper aims to provide a comprehensive view on cardiac fibrosis, with a focus on the main pathophysiological pathways involved in its occurrence and progression, its role in various cardiovascular conditions, on the techniques available for fibrosis identification and quantification, and on the potential of currently available and emerging therapeutic strategies to counteract the development and/or progression of fibrosis in CVDs.

## 2. The Extracellular Matrix—From Physiology to Pathophysiology

The cardiac muscle and the conduction system, supported by a fibrous skeleton, constitute the basic framework of the heart. The cardiac wall consists of three overlapped layers: the epicardium, composed of fibro-elastic and adipose tissue, the myocardium, consisting of cardiomyocytes arranged in layers and surrounded by a complex network of proteins that form the extracellular matrix (ECM), and the endocardium, made up of endothelium and subendothelial connective tissue [3]. At the level of the myocardium, the ECM works as a scaffold for cellular components and contributes to the transmission of the contractile force [4]. Tension strength is mostly provided by thick type I collagen, which accounts for ≈85% of the cardiac collagen, whereas type III collagen, present in smaller amounts (≈11%), is responsible for maintaining the elasticity of the ECM [4]. In addition to collagen, the ECM also contains elastic fibers, fibronectin, glycoproteins, glycosaminoglycans, proteoglycans, latent growth factors, and proteases [4].

### 2.1. Cellular Components Involved in Cardiac Fibrosis

**Figure 1:**
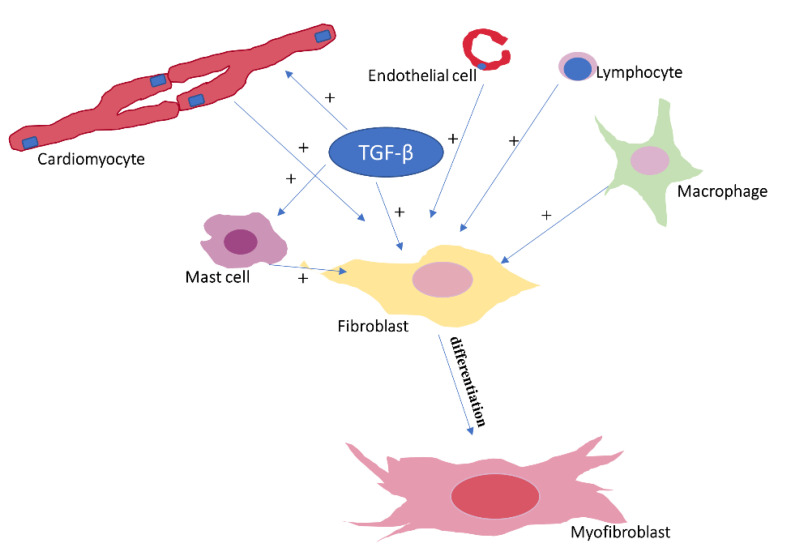
Interactions between different cardiac cells involved in the development of cardiac fibrosis. Cardiac cells (i.e., cardiac myocytes, macrophages, mast cells, lymphocytes, endothelial cells, and fibroblasts) regulate cardiac fibrosis in a coordinated manner. In the presence of cardiac injury, these cells release inflammatory mediators that stimulate fibroblast-to-myofibroblast differentiation, contributing to the development of fibrotic tissue. Transforming growth factor-*beta* (TGF-*β*) is among the most relevant of these profibrotic mediators. “+” designates a stimulatory effect.

The main cellular component involved in cardiac fibrosis is represented by the ***cardiac fibroblasts***, which are responsible for maintaining ECM integrity by regulating collagen turnover [5]. In contrast to cardiomyocytes, fibroblasts are non-excitable cells. Fibroblasts are connected, however, via gap junctions, to the neighboring cardiomyocytes, thereby contributing to optimal electrical conduction within the heart [6]. In settings favoring cardiac fibrosis, such as ischemic, hypertensive, or valvular heart disease, fibroblasts transdifferentiate into ***myofibroblasts*** (Figure 1), hybrid fibroblast/cardiomyocyte cells that express numerous ultrastructural and phenotypic characteristics of muscle cells, but not excitability [7]. Although most myofibroblasts originate in the myocardium, studies have shown that they can also have hematopoietic or endothelial origin [7]. Myofibroblasts play critical roles in both myocardial repair and fibrosis [8] and can be identified in the damaged myocardium already from the early stages of the fibrotic response by highlighting cytoplasmic actin-derived stress fibers and later *α*-smooth muscle actin [9]. Smooth muscle myosin heavy chain, paxillin, and tensin can also be used as myofibroblast biomarkers [10].

***Monocytes*** and ***macrophages*** are also critical for both the initial and the chronic phase of the fibrotic response, but they also contribute to resolution of fibrosis [11]. Their ability to exert both pro- and antifibrotic effects can be explained by the large heterogeneity of these cells, which is related to the presence of several specific cell subpopulations and to their variable response to microenvironmental factors [11]. Certain monocyte and macrophage subpopulations have the ability to differentiate into myofibroblasts and to secrete numerous profibrotic cytokines (such as interleukin (IL)-1*β*, tumor necrosis factor (TNF)-*α*, and IL-6), growth factors (e.g., transforming growth factor *β* (TGF-*β*), platelet-derived growth factors (PDGFs), and fibroblast growth factors (FGFs)), and proteases, thereby participating in ECM remodeling [11]. The removal of dead cells by macrophages via phagocytosis facilitates fibroblasts growth, further contributing to myocardial fibrotic remodeling [11]. In parallel, however, monocytes and macrophages also act to eliminate profibrotic stimuli via phagocytosis of apoptotic myofibroblasts, ECM cells, and residues [11]. Due to their remarkable functional plasticity, macrophages also regulate the secretion of cytokines and growth factors in response to changes in the microenvironment [11]. Other cells of hematopoietic origin, such as the ***mast cells***, have also been shown to play important roles in the fibrotic process related to myocardial infarction and various cardiomyopathies [12]. The role of mast cells in cardiac fibrosis seems to be primarily related to their increased content in granules rich in bioactive mediators, cytokines, and growth factors, including histamine and mast cell-specific proteases tryptase and chymase [12]. Although the role of Th2 ***lymphocytes*** in pulmonary fibrosis has been thoroughly reviewed [13], the involvement of lymphocytes in cardiac fibrosis is much less clear. A profibrotic effect of Th17 cells has been reported in experimental myocardial fibrosis models [14], but other subsets of T lymphocytes have been shown to act as fibrosis inhibitors [15]. ***Neutrophils***, the first cells that arrive at the site of a tissue injury, have also been shown to play critical roles in myocardial inflammation and consequent fibrosis. After acute myocardial infarction, neutrophils accumulate at the border between the healthy and the necrotic tissue and release inflammatory mediators and proteolytic enzymes that degrade necrotic myocardial cells and ECM residues [16]. Neutrophil persistence at the site of the injury appears to also cause, however, additional damage to viable cardiomyocytes [16]. Neutrophil inhibition one week after myocardial infarction has been shown to cause a paradoxical increase in fibroblast activity and excessive collagen deposition [17], suggesting that the moment of such a therapeutic intervention may be critical. Meanwhile, in a myocarditis animal model, neutrophil extracellular traps strongly correlated with the amount of collagen deposited and inhibition of cytokines responsible for neutrophil recruitment attenuated collagen deposition in that model [18].

The angiogenic response and the presence of perivascular fibrosis in settings associated with cardiac fibrosis have drawn attention toward a potential role of ***endothelial cells*** in cardiac remodeling and fibrosis, mainly via the secretion of endothelin, a major promotor of fibrotic matrix production, by these cells [19]. By releasing proinflammatory cytokines and chemokines, the endothelium has an important ability to recruit numerous types of fibrogenic cells [20]. In addition, endothelial cells can take, via mesenchymal transition, a mesenchymal cell phenotype, which enhances their invasiveness and migratory capacity, resistance to apoptosis, and ability to produce ECM components [7]. However, similarly to other types of cells, endothelial cells also possess antifibrotic effects via the secretion of factors such as interferon-γ-inducible protein-10/chemokine (C-X-C motif) ligand 10 and hypoxia inducible factor-1, which have been shown to protect the murine heart and the aorta from pressure overload via suppression of TGF-*β* signaling [21].

***Cardiac myocytes*** can also trigger, through their death, a profibrotic inflammatory response [22]. Moreover, in certain pathological settings, even viable cardiomyocytes can promote, via pannexin-1 channels-induced ATP release, the activation of interstitial fibroblasts [23]. Deoxycorticosterone/salt-sensitive cardiomiocyte mineralocorticoid receptors have also been shown to play important roles in cardiac inflammation and fibrosis, whereas loss of these receptors has been shown to attenuate the cardiac fibrotic response [24]. Cardiomyocyte-selective TGF-*β* receptor II (T*β*RII) blockade decreased interstitial fibrosis in response to pressure overload [25], whereas cardiomyocyte-specific overexpression of angiotensin II (Ang II) type 2 receptor (AT2) was shown to exhibit antifibrotic actions mediated by the activation of the kinin–nitric oxide system [26].

### 2.2. Extracellular Components Involved in Cardiac Fibrosis

The accumulation of excessive amounts of fibrillar and non-fibrillar ***collagen*** within the myocardium represents the landmark of cardiac fibrosis [27,28]. In the remodeled heart, fibrillar collagen is represented by type I and type III collagen, the ratio between the two depending on the context that favored cardiac fibrosis development [27,28]. Cardiac myofibroblasts are the main source of cardiac collagen. Once secreted, collagen is assembled and cross-linked into a network that provides mechanical support and structural integrity to bear the increased stress and load in the presence of myocardial injury [27,28]. Non-fibrillar collagen type IV, VI, and VIII is also present in cardiac fibrosis [29]. Of these, type VI collagen has been shown to activate cardiac fibroblasts and promote myofibroblast conversion, whereas its absence has been associated with a reduction in myocardial fibrosis [29]. Other ECM components, such as amino- and proteoglycans, elastin, fibronectin, and laminin, are also present, and they play critical roles in maintaining cardiac structural integrity. Elastin provides resilience and elasticity to the cardiac wall, fibronectin fibers, organized in a fibrillar network at the cell surface, influence the structural and mechanical properties of the ECM, whereas the structural role of laminin translates into ECM cells anchoring and binding to multiple other proteins present within the matrix [4].

### 2.3. Types of Cardiac Fibrosis

The ability of the cardiac muscle to regenerate in response to injury is extremely low. Cardiac repair therefore occurs mainly via fibroblasts activation and differentiation into myofibroblasts, which is followed by excessive collagen deposition, fibrosis, increased ECM stiffness, and impaired cardiac contractile function [30]. Three major types of fibrotic changes have been described in the heart: replacement, interstitial, and perivascular fibrosis [30]. Replacement fibrosis is characterized by the loss and consequent fibrotic replacement of cardiac myocytes. Interstitial fibrosis includes two subtypes: reactive and infiltrative fibrosis. Reactive fibrosis occurs in response to pressure overload and is characterized by excessive ECM, without significant loss of cardiomyocytes, whereas the accumulation of insoluble proteins in the heart cells, as seen in Fabry disease, is defined as infiltrative fibrosis [30]. Finally, perivascular fibrosis involves the deposition of connective tissue around the blood vessels, as often seen in patients with hypertensive heart disease [30].

Regardless of the underlying cause, the accumulation of ECM proteins within the cardiac interstitium initially occurs as a beneficial, protective mechanism that promotes wound healing and tissue regeneration. Later, alterations in ECM composition and quality lead to fibrosis progression beyond the physiological threshold and to negative consequences on myocardial excitation–contraction coupling [31]. The distorted cardiac architecture increases ventricular stiffness and alters the contraction and relaxation of the heart, resulting in cardiac systolic and diastolic dysfunction [31]. Concomitantly, fibrosis disturbs the normal electrical activity of the heart, promoting both brady- and tachyarrhythmias [32]. Whereas conduction blocks in the sinoatrial and/or atrioventricular nodes caused by fibrosis favor bradyarrhythmias, tachyarrhythmias often occur due to the increased propensity to re-entry of the fibrotic myocardium [32].

### 2.4. Molecular Pathways of Myocardial Fibrosis

**Figure 2:**
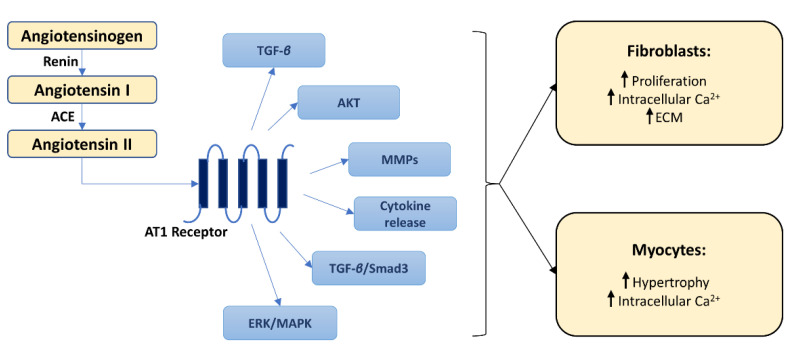
Pathways related to angiotensin II and their contribution to myocardial fibrosis. The figure describes the formation of angiotensin II (left part of the figure) and the consequent activation, via AT1 receptors, of numerous inflammatory and profibrotic pathways (middle part of the figure), which will eventually lead to profibrotic cardiac fibroblast and myocyte changes (right part of the figure). Myocyte hypertrophy has been shown to promote fibrosis by stimulating fibroblast activation via a complex network of downstream signal transduction pathways and by increasing the production of growth factors. “↑” designates an increase in profibrotic cardiac fibroblast and myocyte changes. ACE—angiotensin-converting enzyme; AKT—protein kinase B; AT1—angiotensin II type 1 receptor; ECM—extracellular matrix; ERK—extracellular signal-regulated kinase; MAPK—mitogen-activated protein kinase; MMPs—matrix metalloproteinases; TAK1—TGF-*β*-activated kinase 1; TGF-*β*—transforming growth factor-*beta*.

Extensive evidence links the activation of the ***renin–angiotensin–aldosterone system*** (RAAS) with the pathogenesis of cardiac fibrosis. The main effector of this system, Ang II, has a wide range of cardiac physiological and pathophysiological effects [33]. Cells present in the heart, particularly macrophages and fibroblasts, have been shown to produce both renin and the angiotensin-converting enzyme (ACE), which are required to generate Ang II. Once released, Ang II stimulates cardiac fibroblasts, directly and indirectly (via TGF-*β*), promoting collagen production by these cells (Figure 2) [34]. In parallel, AngII decreases the activity of matrix metalloproteinase (MMP)-1, thereby concomitantly reducing collagen degradation [33]. These profibrotic effects of Ang II occur mainly via the AT1 receptors and multiple subsequent intracellular signaling pathways [33]. Among them, the mitogen-activated protein kinase (MAPK) and the phosphoinositol-3 kinase/Akt pathways have been shown to regulate cardiac cells survival, apoptosis, and growth and to play critical roles in Ang II-induced cardiac remodeling [35,36].

In contrast, AT2 receptor stimulation counteracts the profibrotic effects of AT1 by suppressing fibroblast proliferation and matrix synthesis [37]. Another component of the RAAS system, aldosterone, also contributes to excessive ECM accumulation by activating macrophages, cardiac myocytes and fibroblasts and increasing the expression of proinflammatory cytokines and chemokines [38].

**Figure 3:**
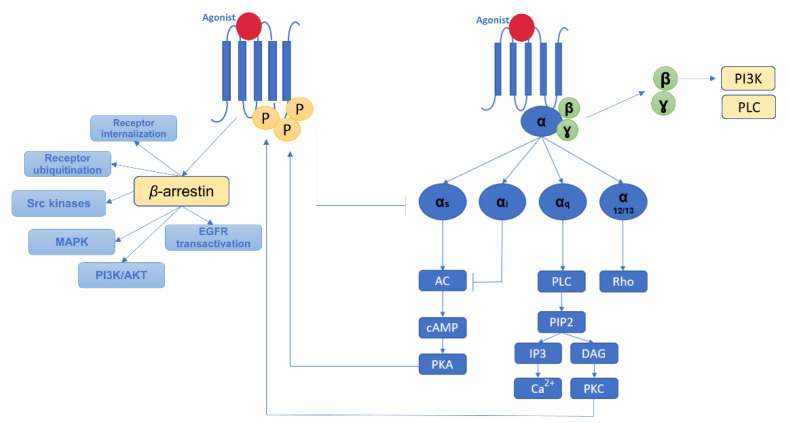
G protein-coupled receptors-related pathways and *β*-arrestin-mediated events. G-protein coupled receptors are transmembrane proteins embedded in the membrane of cardiomyocytes, fibroblasts, endothelial, and vascular smooth muscle cells that convert extracellular signals into intracellular responses. When activated by agonists (e.g., epinephrine, peptide hormones), inactive G protein heterotrimers dissociate into separate, active G*α* and G*βγ* subunits that differentially control downstream signal transduction. Intracellular mediators such as protein kinases A and C resulted from this process further phosphorylate the receptors and activate *β*-arrestin-mediated signaling, activating subsequent signaling cascades involved in cardiac fibrotic disease. AC—adenylyl cyclase; AKT—protein kinase B; cAMP—cyclic adenosine monophosphate; DAG—diacylglycerol; EGFR—epidermal growth factor receptor; IP3—inositol trisphosphate; MAPK—mitogen-activated protein kinase; PI3K—phosphoinositide 3-kinase; PIP2—phosphatidylinositol-4,5-bisphosphate; PKA—protein kinase A; PLC—phospholipase C; PKC—protein kinase C.

***G protein-coupled receptors*** (GPCRs) are cellular receptors that activate G-protein-dependent intracellular signaling pathways [39]. In parallel, several GPCR kinase- and *β*-arrestin2-mediated processes act as regulatory mechanisms to prevent excessive G protein activation (Figure 3) [39].

Several ‘biased ligands’ can activate signaling pathways independent of the G proteins but dependent on GPCR kinase/*β*-arrestin2 [39]. One such ‘biased ligand’ is metoprolol, which can therefore promote cardiac fibrosis and alter the cardiac diastolic function [39]. *Beta*-adrenergic receptors (*β*ARs) are the predominant GPCR subtype expressed within the heart [40]. In physiological conditions, *β*1ARs represent ≈80% of the total cardiac *β*ARs. However, in the setting of heart failure, *β*1ARs percentage can decrease to as low as 60%, with a concomitant increase in the proportion of *β*2ARs, whereas *β*3ARs are present in the heart in much smaller amounts [40]. Excessive *β*1ARs stimulation has been linked with myocyte apoptosis [40]. Meanwhile, the role of *β*2ARs in this setting remains controversial. Some studies suggested that *β*2Ars-mediated signaling could be cardioprotective [40], but in others, non-specific *β*ARs stimulation with isoproterenol and transgenic overexpression of *β*2ARs were highly profibrotic [41,42], leaving this topic an open area for future research. The role of *β*3AR in cardiac fibrosis remains even less understood, although recent studies suggest that *β*3AR-mediated signaling could modulate oxidative stress-dependent paracrine signaling and consequently exhibit antifibrotic effects [43].

***Endothelin-1*** (ET-1), a protein synthesized by the vascular endothelium, has also been shown to play key roles in cardiac remodeling and dysfunction by promoting ECM synthesis and decreasing collagenase activity [44]. In addition, in vitro studies have shown that ET-1 increases fibroblasts’ resistance to apoptosis [45], whereas ET-1 antagonization has been shown to attenuate cardiac fibrosis related to hypertension and myocardial infarction [46].

Immediately after myocardial injury, inflammatory cells, fibroblasts, and cardiomyocytes release a vast amount of cytokines and growth factors [11]. Among them, ***TNF-α***, ***IL-1β***, and ***IL-6*** levels are particularly increased in response to the inflammatory process and strongly contribute to the future development of cardiac fibrosis [47]. The role of TNF-*α* in cardiac fibrosis is supported by numerous experimental and clinical studies [48,49]. Meanwhile, the absence of TNF-*α* reduced the inflammatory response and cardiac fibrosis in mice [47], and TNF-*α* inhibition has been shown to improve left ventricular structure and function in patients with advanced heart failure [48]. In contrast to the vast majority of profibrotic stimuli, TNF-*α* does not exert its fibrotic effect by an increase but rather by a decrease in collagen synthesis, suggesting that the profibrotic effect of TNF-*α* is more likely to occur as a response to ECM degradation [49]. Additional mechanisms involved in TNF-*α*-induced fibrosis include synthesis of the matrix protein cellular communication network factor 4, favoring fibroblast proliferation, increased TGF-*β*1 expression, immune cell activation and proliferation, and promotion of AT1 receptors synthesis [50]. Data regarding the role of IL-1*β* in cardiac fibrosis are rather controversial. Some experimental studies suggested that IL-1*β* may promote cardiac fibroblast migration, while others reported the opposite [51]. In patients with rheumatoid arthritis, IL-1 inhibition led, however, to a significant improvement in left ventricular function [52]. In some, but not all studies, a relationship was found between low IL-6 levels and cardiac fibrosis [53,54].

**Figure 4:**
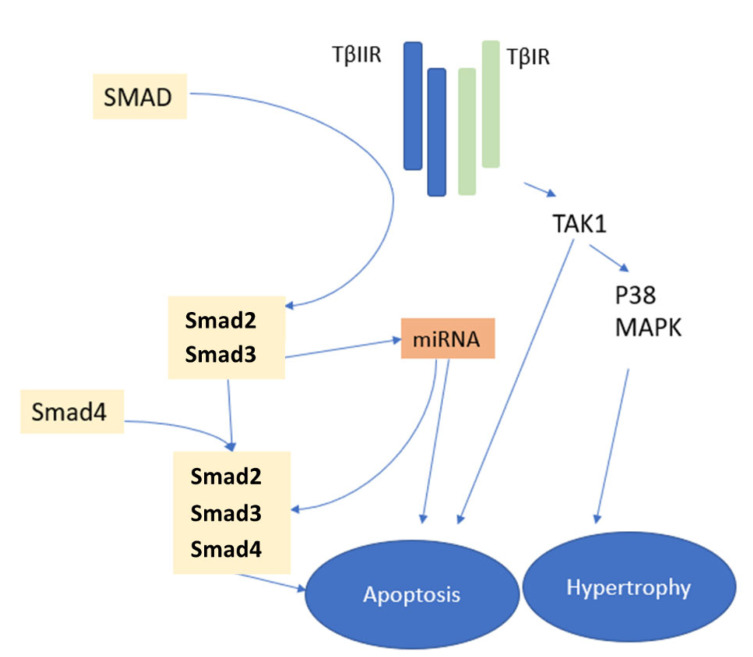
Transforming growth factor *beta*-related pathways and their contribution to myocardial fibrosis. Paracrine factors in fibroblasts, the most important of which is transforming growth factor-*beta*, induce profibrotic responses in cardiomyocytes. The activation of type I and II transforming growth factor-*beta* receptors regulates cell phenotypes by activating Smad- and non-Smad-related signaling pathways that eventually result in cardiomyocyte apoptosis and hypertrophy. MAPK—mitogen-activated protein kinase; TAK1—transforming growth factor-*beta*-activated kinase 1; T*β*IR—transforming growth factor-*beta* receptor type I; T*β*IIR—transforming growth factor-*beta* receptor type II.

The most studied fibrosis-related growth factor, ***TGF-β***, has been shown to play a central role in maladaptive cardiac remodeling in both myocardial infarction and heart failure (Figure 1) [55,56]. In gain-of-function studies, cardiac overexpression of TGF-*β*1 increased collagen deposition and promoted cardiac fibrosis, whereas TGF-*β*1 deficiency has been associated with a lower degree of aging-related cardiac fibrosis [55,57]. The stimulating effect of TGF-*β* on cardiac fibroblasts appears to be the basis for an increased synthesis of ECM proteins [57]. Its effects on monocytes, lymphocytes, and cardiomyocytes further contribute to the profibrotic effects of TGF-*β* [58] as well as the reversal of the fibrosis degradation/preservation balance toward a matrix-preserving pattern via inhibition of collagenases and induction of protease inhibitors such as plasminogen activator inhibitor-1 and tissue inhibitors of metalloproteinases [57]. Meanwhile, in experimental studies, a loss of TGF-*β* receptors reduced cardiac fibrosis [59], further supporting the importance of TGF-*β* signaling cascades in cardiac fibrosis. The TGF-*β* signaling cascades exert their profibrotic effects through Smads, intracellular effector proteins, but also through Smad-independent pathways, both leading to fibroblast activation (Figure 4) [60].

Studies have shown Smad3 signaling to be critically involved in chronic fibrotic cardiac remodeling and to contribute to fibroblasts activation, *α*-smooth muscle actin expression, and synthesis of ECM [61]. In contrast, myofibroblast-specific Smad2 signaling appears to be only transiently implicated in early adverse remodeling and does not seem to play a major role in fibroblast activation [60]. Smad-independent profibrotic TGF-*β*-related pathways involve p38, MAPK, extracellular signal-regulated kinase, and TGF-*β*-activated kinase 1 (TAK1) signaling pathways activation (Figure 1) [62].

***Exosomes*** are extracellular microvesicles that supply cells with RNA, proteins, lipids, and other biologically active signaling molecules, while acting as couriers for intercellular communication. Recent evidence indicates that in the presence of cardiac fibrosis, there is altered intercellular communication via the exosomes [63]. In addition, exosomes have been shown to alter the process of cardiac repair and to cause fibrosis via the modulation of fibroblast function [64]. During cardiac injury, exosomes promote the activation of naive fibroblasts to initiate the wound-healing process and contribute to fibroblast differentiation into myofibroblasts [64]. Injured endothelial cells have also been shown to secrete exosomes enriched with profibrotic, antiangiogenic factors, and microRNAs that will further contribute to cardiac fibrosis [65].

## 3. Identification and Quantification of Myocardial Fibrosis

Invasive and non-invasive methods have been developed over time in order to identify and quantify myocardial fibrosis (Table 1). Of these, myocardial biopsy is the most reliable method.

### 3.1. Invasive Methods to Investigate Cardiac Fibrosis

A histological evaluation of myocardial tissue samples obtained during myomectomy, open heart surgery, or endocardial biopsy remains the gold standard for diagnosing and quantifying myocardial fibrosis [66]. Using appropriate staining methods, histological analysis of the collagen volume fraction (CVF; i.e., the ratio of the sum of connective tissue areas to the sum of all areas of connective and muscle tissue) is the most widely used method for quantitative evaluation of cardiac fibrosis [66]. No cut-off values have been defined so far; however, for CVF, the values varying considerably from one study to another. In addition, the use of this technique is strongly limited by the fact that it requires direct, invasive access to cardiac tissue samples, which carries inevitably the risk of several potentially major complications. Moreover, in settings with regional fibrosis, obtaining tissue samples does not guarantee a correct diagnosis.

### 3.2. Non-Invasive Methods to Evaluate Cardiac Fibrosis

Several ***blood biomarkers*** have been proposed to assess cellular and molecular changes that reflect the amount of fibrotic tissue of the heart. C-terminal propeptides of collagen I and N-terminal propeptides of collagen III highly correlated with total CVF, creating optimism about future clinical use [67]. However, both biomarkers have low specificity, and increased levels can also be observed in liver fibrosis [68]. High levels of galectin-3, a molecule that accelerates fibrosis by stimulating myofibroblast activation, have been associated with increased mortality and worse prognosis in heart failure. However, no associations were found between galectin-3 levels and CVF [69]. Circulating levels of miR-21, one of the regulators of fibroblast activity, have been shown to correlate with myocardial expression of type I collagen mRNA [70]. Its potential use as a blood biomarker for CVF and its cut-off values remain, however, questionable. Higher TGF-*β* levels have been reported in patients with heart failure compared with control [71]. However, its correlation with CVF remains to date unclear.

***Cardiac magnetic resonance imaging*** (MRI) with T1 relaxometry can provide rapid information on cardiac edema, fibrosis, and deposition diseases, whereas replacement fibrosis can be evaluated using gadolinium MRI [72]. It should be noted, however, that this latter technique is not reliable in settings characterized by diffuse fibrosis in a homogeneous myocardium, although MRI-quantified fibrosis did correlate with cardiac function in patients with heart failure with preserved ejection fraction [73]. In the absence of edema or infiltrative disease, calculating the extracellular cardiac volume by T1 relaxometry after gadolinium injection allows evaluating even small amounts of fibrosis, and the results have been shown to correlate better with biopsy results than those obtained using other MRI-based techniques [72].

With its favorable safety profile and relative ease of use, ***echocardiography*** is often the first investigation used for assessing myocardial function and structure and for obtaining indirect data on cardiac fibrosis. Using speckle tracking echocardiography, one can quantify myocardial thickening, shortening, and rotation dynamics [74]. In hypertrophic cardiomyopathy, regional impairment of myocardial function assessed by speckle tracking echocardiography correlated with the presence of fibrosis detected by MRI [75]. Echocardiographic measurement of calibrated integrated backscatter is a technique developed to quantify the ultrasonic reflectivity of the myocardium in relation to the high reflectivity of the pericardium and the low reflectivity of blood [76]. In patients with dilated or hypertrophic cardiomyopathy and extensive fibrosis, the results have been shown to correlate significantly with the amount of myocardial fibrosis measured histologically [76]. The most important limitation of all echocardiographic methods remains, however, the need to obtain high-quality images.

## 4. Targeting Myocardial Fibrosis—A Magic Pill in Cardiovascular Medicine?

Immediately after any cardiac injury, a dynamic process of remodeling is initiated that is critical for stabilization of the cardiac wall. Expansion of non-contractile, collagen-rich tissue will lead, however, not only to scar tissue maturation but also to progressive adverse remodeling, which stands at the foundation of many CVDs. Targeting myocardial fibrosis could therefore provide tremendous benefits in many CVDs. However, because of the critical role that fibrosis plays in wound healing and tissue repair, concerns remain that fibrosis manipulation strategies may not completely innocuous. One of the major goals of novel fibrosis-oriented therapies is therefore not to withhold the process of fibrosis but rather to modify the properties of the scar tissue and to direct fibrotic pathways toward the formation of a functionally efficient fibrotic tissue.

### 4.1. Cardiac Antifibrotic Effects of Non-Antifibrotic Drugs

Clinical and experimental studies have shown that for numerous drugs created for various, non-antifibrotic purposes, the benefit could be at least partially linked to their antifibrotic effects (Table 2).

Given the major role that ***RAAS*** plays in cardiac fibrosis pathogenesis, molecules that act at different RAAS levels have been investigated for their potential antifibrotic effects (Table 2). Aliskiren, a molecule that binds to renin and limits the initial step required for Ang II synthesis, has been shown to limit myocardial collagen deposition via Ang II-dependent and (pro)renin receptor-related pathways [90]. Already used as first-line therapy in a vast majority of CVDs, ACE inhibitors were also shown to reduce myocardial fibrosis in several animal models [33]. The decrease in myocardial collagen content induced by ACE inhibitors was related to a significant decrease in type I (but not type III) collagen as well as to an increase in gelatinase activity [91]. However, several clinical trials have failed to associate ACE inhibitors with a reduction in hospitalization and mortality in patients with various conditions characterized by extensive cardiac fibrosis, suggesting that ACE inhibition may be insufficient to effectively block the activity of multiple fibrosis pathways [92]. The blockade of Ang II AT1 receptors efficiently reduced fibrosis in both clinical and experimental settings [79,93]. Independently of their antihypertensive effects, AT1 receptor inhibitors have been associated with a more important reduction in type I collagen than ACE inhibitors [91]. Aldosterone inhibition reduced ECM, decreased fibrotic markers levels, significantly improved ventricular function in animal studies, and significantly reduced mortality in patients with heart failure and reduced ejection fraction [94]. Although in the Treatment of Preserved Cardiac Function Heart Failure With an Aldosterone Antagonist (TOPCAT) trial aldosterone inhibition failed to significantly improve the composite endpoint of cardiovascular death, aborted cardiac arrest, or hospitalization [95], a post hoc analysis of the trial indicated that this strategy may improve symptoms and hospitalization in certain patient subgroups [96].

***Beta-blockers*** have been shown to prevent fibrosis and improve survival in animal models and to favorably affect prognosis in heart failure patients with preserved ejection fraction [97,98]. However, in humans, the antifibrotic effects of *beta*-blockers remain highly controversial (Table 2) [99]. This discordance could be at least partly related to the type of *beta*-blocker administered; there are studies suggesting that different *beta*-blockers may have opposite effects on the development of cardiac fibrosis [37]. ***Calcium channels blockers*** have also been shown to reduce cardiac fibrosis in different animal studies. The long-term administration of mibefradil, verapamil, and amlodipine reduced adverse cardiac remodeling and improved ventricular function in rats with ischemic heart failure [100]. In humans, the calcium channel blocker tetrandrine prevented myofibroblast activation and reduced cardiac fibrosis via a mechanism independent of calcium channel blockade and of the reduction in hemodynamic load [101]. Despite their anti-inflammatory effects, the ability of ***statins*** to alleviate cardiac fibrosis remains questionable. In rats with hypertensive heart disease, statin therapy reduced adverse cardiac remodeling, ventricular dysfunction, and progression to heart failure [102]. In patients with heart failure, statins reduced type III procollagen [81], the amount of myocardial fibrotic tissue and plasma markers of fibrosis [103]. However, in a 6-month randomized placebo-controlled study, the effect of statins on cardiac remodeling was neutral [104], whereas in another study, statin use was associated with an increase in serum collagen markers [82]. In experimental studies, ***endothelin inhibitors*** reduced fibrosis in multiple organs, attenuated cardiac remodeling, and significantly increased survival [105]. This did not seem to be the case, however, in patients with heart failure [106]. Adding endothelin antagonists to ACE inhibitors, *beta*-blockers, or aldosterone antagonists also does not seem to provide additional benefits in terms of cardiac remodeling in patients with heart failure [107].

### 4.2. Novel Targets for Cardiac Fibrosis Prevention and Therapy

Studies have identified a number of novel targets for cardiac fibrosis prevention and therapy (Table 3) [108].

Due to its major role in cardiac fibrosis, the ***TGF-β signaling pathway*** has become one of the most tempting targets in this setting. Anti-TGF-*β* antibodies were shown to efficiently decrease fibroblast activation and to improve diastolic function in rats with cardiac pressure overload, although they did not provide any improvement in myocyte hypertrophy or systolic function in those rats [109]. Moreover, in an experimental model of myocardial infarction, although anti-TGF-*β1* antibodies reduced fibrosis, their use was associated with increased mortality and dilatation of the left ventricle [110]. Alternative approaches, such as soluble T*β*RII, a competitive inhibitor of TGF-*β*, and inhibitors of the TGF*β*RI kinase (ALK5) have also been investigated. Both strategies reduced collagen synthesis and cardiac fibrosis but also manifested non-negligible side effects [111,112]. GW788388, a blocker of both ALK5 and TGF*β*RII that has improved pharmacokinetics and minimal toxic effects, reduced left ventricular remodeling in rats with myocardial infarction [113], but the full effects of this agent remain to be established. Inhibition of TAK1 and p38-MAPK has also been investigated, and both showed promising effects on cardiac fibrosis [114,115]. Pirfenidone and tranilast, two other TGF-*β* inhibitors, have also shown promising results in preclinical studies [116,117]. For tranilast, clinical data on cardiac fibrosis are still lacking. Meanwhile, in a recent phase II clinical trial, pirfenidone significantly reduced myocardial fibrosis in patients with heart failure and low ejection fraction without causing serious adverse cardiac events [83]. Pirfenidone’s numerous extra-cardiac, including gastrointestinal, neurological, and dermatological side effects [118], remain, however, a serious concern. In mice, both Smad3 deficiency and Smad3 inhibition efficiently reduced the amount of cardiac fibrosis and prevented the decline in left ventricular ejection fraction [119], pointing Smad3 inhibition as a promising new antifibrotic approach. Increased levels of endogline, a co-receptor of TGF-*β*1 and TGF-*β*3, have been associated with both heart failure and acute myocardial infarction [120]. Meanwhile, decreased endoglin expression reduced the amount of cardiac fibrosis and improved survival in mice with heart failure [121]. The antifibrotic effect of blocking other TGF-*β*-related pathways, such as the RhoA–serum response factor-myocardin-related transcription factor [122] or the transient canonical potential receptor channels pathways [123] also remains to be evaluated in future studies.

In cardiac myocytes, the ***PDGF*** family includes PDGF-A and -C, while PDGFR*α*-positive cells have been described in the cardiac interstitium. Endothelial cells express PDGF-B and -D, while pericytes and smooth muscle cells are PDGFR*β*-positive [124]. All PDGFs have been shown to play a role in cardiac fibrosis. In transgenic mice, the overexpression of PDGF-C and -D has been associated with cardiac fibrosis and hypertrophy [125]. Meanwhile, PDGF blockade reduced interstitial fibrosis in rats with myocardial infarction and decreased fibroblast activation in dogs [126], and neutralizing PDGF receptor-specific antibodies suppressed cell proliferation and collagen expression in cardiac fibroblasts [127].

Elevated levels of connective tissue growth factor (***CTGF***) have been detected in myocardial infarction and heart failure and have been shown to correlate with the degree of myocardial fibrosis [128]. The profibrotic effects of CTGF appear to emerge from its ability to stimulate fibroblasts’ proliferation and transformation into myofibroblasts, although its potential to intrinsically induce fibrosis seems to be rather low [129]. In some experimental studies, the overexpression of CTGF had no significant profibrotic effects [130]. Other studies indicated, however, that CTGF can exert profibrotic effects [131], and that CTGF inhibition with monoclonal antibodies enhances cardiac repair, limits fibrosis, and ensures better preservation of left ventricular systolic function post-myocardial infarction [132].

Although the exact mechanisms remain insufficiently understood, the administration of ***angiotensin receptor–neprilysin inhibitors*** (ARNIs), a drug complex composed of a neprilysin inhibitor precursor and a non-peptide Ang II receptor blocker, has been shown to decrease the risk of death and hospitalizations in heart failure patients [133]. Preclinical studies have shown significant improvement in ventricular remodeling following neprilysin inhibition, and clinical trials later confirmed these results in patients with heart failure treated with ARNIs [134,135]. The benefit appears to emerge from the synergistic actions of the two components of ARNIs on multiple mechanisms involved in pathological cardiac remodeling. Neprilysin inhibition increases the concentrations of vasodilator peptides, such as the atrial and brain natriuretic peptides, and bradykinin, thereby improving myocardial perfusion in the infarcted area, but also increases concomitantly the concentrations of Ang II, whose effects are efficiently counteracted by the Ang II receptor blocker [135,136]. According to preclinical data, cardiac fibrosis and adverse remodeling are counteracted by ARNIs mainly via inhibition of the Wnt/*β*-catenin pathway [137]. In addition, ARNIs appear to attenuate cardiomyocyte growth and to increase the capillary/cardiomyocyte ratio at the level of the border area between the infarcted and the healthy myocardium [135], and even to reduce myocardial fibrosis, as reflected by the reduction in MMP-2, MMP-9, and N-terminal propeptide of type I procollagen, leading to a reduction in left atrial size and to significant improvement in left ventricular ejection in patients with heart failure [138,139,140]. Disappointingly, however, ARNIs failed to reduce hospitalizations and cardiovascular death in patients with heart failure and a left ventricular ejection fraction ≥45% (Table 2) [141].

### 4.3. Targeted Blockade—Aiming to Obtain a ‘Better Scar’

Although the direct manipulation of mechanisms involved in fibroblast recruitment is not currently regarded as a primary target in the management of CVDs, this strategy carries a major potential to favorably influence scar formation and tissue remodeling.

Monocyte chemoattractant protein-1 (***MCP-1***) provides key signals for the migration and infiltration of inflammatory cells and activated fibroblasts. The overexpression of MCP-1 at the cardiac level promotes fibroblast accumulation, contributing to improved cardiac function and myocardial remodeling in transgenic myocardial infarction mice [142]. Meanwhile, MCP-1 deletion significantly reduced Ang-II-induced fibrosis by reducing the uptake and differentiation of CD45+ fibroblast precursors [143]. The manipulation of MCP-1 could thus emerge as a promising strategy to influence progenitor fibroblast cells and to prevent fibrosis and adverse cardiac remodeling.

***Modulation of collagen accumulation and maturation*** in order to obtain a myocardial collagen network adapted to the local mechanical conditions could represent another potential target in fibrosis-related CVDs. In myocardial infarction, expansion of the infarcted area is associated with poor mechanics and increased risk of rupture of the injured wall. Approaches that stimulate compaction of the infarcted area by increasing collagen cross-linking inside the scar could thus provide an option to counteract maladaptive cardiac fibrosis. Modulation of lysyl oxidases, enzymes produced by activated fibroblasts that stiffen the collagen network by boosting collagen fibers cross-linking, appears to be particularly promising in this regard [144].

***Modulation of cardiomyocyte-fibroblast coupling*** inside the scar area, while keeping the outer area unchanged may also help to create a ‘better scar’. In myocardial infarction, this would translate into increased trans-scar communication and transformation of the infarcted area into an ‘electrically-transparent scar’, with more homogeneous electrical activity and lower risk of re-entry [145]. This could be obtained by upregulating heterotypic connexin (Cx)-coupling with drugs such as rotigaptide, which significantly enhanced metabolic coupling in Cx43-coupled cells and attenuated gap junction closure under metabolic stress [146].

***Fibroblast-derived microRNA-enriched exosomes***, paracrine signaling mediators of cardiac hypertrophy and remodeling, are also regarded as highly promising. In vivo silencing of miR-21 reduced fibrosis in pressure-overload-induced disease and increased survival following myocardial infarction [70]. Other in vivo and in vitro studies suggested that miR-125b promotes profibrotic signaling in endothelial-to-mesenchymal transition and fibroblast activation [147]. miR-29 downregulation has also been associated with increased cardiac fibrosis, while miR-29 overexpression reduced collagen expression in myocardial infarction models [148]. In a mouse model of ATII-induced hypertension, mimetic miR-29 transfection also reduced the development of cardiac fibrosis via the TGF-*β*/Smad3 pathway [149]. More recently, miR-145, miR-30, and miR-133 have also been shown to modulate collagen deposition and to control structural ECM changes [150,151]. However, challenges in targeting microRNAs to prevent cardiac fibrosis remain, which are mainly related to their broad and non-specific effects. Nevertheless, ongoing efforts to identify the molecular targets of non-coding RNAs are promising for future clinical interventions.

***Periostin*** targeting is also seen as a potential option in fibrosis-related CVDs. Periostin acts as a regulator of cardiac fibrosis by altering the deposition, diameter, and cross-linking of collagen fibers, by modifying the mechanical adhesion between fibroblasts and myocytes [152], and by recruiting activated fibroblasts via FAK-integrin signaling [153]. In heart failure patients, periostin distribution and expression has been associated with the amount of fibrotic tissue, suggesting that periostin may be a potential biomarker of cardiac remodeling in this setting [153]. In post-myocardial infarction mice, the genetic manipulation of periostin was shown to improve cardiac function. However, it also led to an overall increase in fibrosis [151]. Thus, the use of periostin as a potential target remains a sensitive issue.

### 4.4. Indirect Blockade of Fibrosis via Stimulation of Myocardial Regeneration/Repair

The targeted delivery of biomaterials composed of natural (e.g., naturally derived matrices) or synthetic (e.g., poly [N-isopropyl acrylamide]-based hydrogels) biomaterial +/− cells or growth factors has been investigated as a potential novel antifibrotic therapeutic strategy with promising results in rodent and large animal models [154]. Decellularized cardiac ECM alone can also be used as a biomaterial to control cardiac fibrosis and to provide support for the infarcted wall [154]. Transcatheter injection of processed decellularized cardiac ECM hydrogel has been shown to promote stem cells recruitment, proliferation, and differentiation into cardiac cells [155] and to be safe for administration in human patients [156]. Although the trial was not designed to assess efficacy, there was a decrease in heart failure symptoms, an increase in 6-min walk test distance, and an improvement in left ventricular remodeling in post-myocardial infarction patients [156]. Acellular patches that provide cells with tissue-specific biochemical cues important for cell migration and differentiation and tissue regeneration have also been investigated. The most used biomaterials include growth factors, ECM molecules, heparin, and thrombomodulin, which help to ensure a uniform surface coating of the polymeric cardiovascular scaffold [157]. The vascular endothelial growth factor, the insulin-like growth factor 1, the hepatocyte growth factor, the myeloid-derived growth factor, neuregulin 1, the epidermal growth factor, and the fibroblast growth factor are the most widely employed to improve the bioresponsive properties of the scaffolds [157].

Cardiac patches that use collagen as a scaffold have also been studied in combination with a variety of cell types capable of exerting paracrine effects or to directly regenerate the injured myocardium [158], whereas fibrin cardiac patches improved cell delivery in a porcine model of post-infarction left ventricular remodeling [159]. Cells that promote adipose-derived stem cell regeneration embedded into platelet-rich fibrin and patched in myocardial infarction rats significantly decreased fibrotic mediators’ levels and increased the expression of antifibrotic markers [160]. The implantation of pluripotent stem cells-derived cardiomyocytes placed on collagen scaffolds into dilated mouse hearts was also shown to decrease cardiac fibrosis and to increase the expression of osteopontin, which is an acidic phosphoglycoprotein that regulates the MMPs [161]. Multiple experimental studies provided highly promising results, and there are several ongoing clinical trials that test the localized delivery of biomaterials and antifibrotic agents.

Cell-sheet implantation has been shown to attenuate remodeling, restore the damaged myocardium, and improve cardiac function in several experimental models of myocardial infarction and dilated cardiomyopathy [162]. The method was tested in patients with myocardial infarction and dilated cardiomyopathy in a phase I clinical trial [163]. Although not adequately powered, the trial indicated a decrease in pulmonary pressure and resistance, as well as in the levels of BNP, an increase in walking distances on the 6-min walk test, and an improvement in the New York Heart Association classification in the treated patients, particularly in those with ischemic heart disease [163].

### 4.5. Modulation of Collagen Turnover

Procollagen processing by procollagen C-proteinase(s) is critical for the maturation of soluble collagen precursors into insoluble collagen and is potentiated by procollagen C-proteinase enhancers (PCPE-1 and -2) [164]. The expression levels of these later proteins have been shown to strongly correlate with the degree of fibrosis in different animal models [164]. The effect of PCPE-1 manipulation on cardiac fibrosis has not been evaluated to date. In the mouse liver, PCPE-1 deficiency decreased, however, the amount of fibrosis [165]. Meanwhile, PCPE-2 null hearts have been associated with a decrease in CVF and with lower myocardial stiffness in mice with aortic constriction [166].

Increased collagen production via Smad7 is amidst the many mechanisms by which miR-21 promotes cardiac fibrosis [167]. Elevated miR-21 expression has been shown to negatively affect collagen cross-linking and, implicitly, CVF [168], suggesting that miR-21 silencing could inhibit collagen synthesis and could thus exhibit antifibrotic effects.

Serelaxin is a recombinant form of human relaxin-2, which is a hormone that contributes, among others, to the degradation of the ECM. The antifibrotic effect of relaxin has been reported in both the kidney and the heart [169] and appears to rely on the prevention of cardiac fibroblast-to-myofibroblast transition via TGF-*β*/Smad3 pathway inhibition [170]. In addition, serelaxin has been shown to be safe in patients with acute heart failure [171], making this molecule particularly appealing for future clinical research.

Alterations in the balance between MMPs and their specific tissue inhibitors (i.e., TIMPs) have been incriminated as contributors to the abnormal production of ECM [172]. Cardiac expression of TIMP-1 and -2 was shown to be significantly increased and strongly correlated with the amount of cardiac fibrosis in patients with pressure overload [172]. Meanwhile, TIMP-3 has been shown to possess an increased affinity for ECM glycosaminoglycans and to alter the fibroblast phenotype [173]. The targeted administration of TIMP-3 could thus emerge as a promising collagen-decreasing strategy. In myocardial infarction pigs, the regional delivery of exogenous TIMP-3 showed positive effects on left ventricular ejection fraction and volume as well as on the extent of the infarcted area [173].

## 5. Gaps in Knowledge, Ongoing and Future Research

Cardiac fibrosis is a complex syndrome that affects not only the structure but also the function of the heart, suggesting that myocardial ECM homeostasis is essential for normal cardiac functioning. Identifying widely available, inexpensive, non-invasive, and highly accurate biomarkers for in vivo quantification of not only gross but also subtle cardiac fibrosis should continue to represent a major priority, as is the case in numerous other clinical settings [174,175]. Multiple strategies have been shown to efficiently counteract fibrosis. However, incomplete knowledge regarding the complex pathogenesis of fibrosis limits advancement in this field. Understanding the activation of cardiac fibroblasts and their role in cardiac fibrosis is necessary to improve our pharmaceutical arsenal. The development of safe and effective antifibrotic strategies also depends on a detailed decipherment of the pathways involved in the antifibrotic response. The window of therapeutic opportunity also remains unknown, at present, both spatially and temporally. Cardiac fibroblasts may respond differently to different therapeutic interventions, depending on the underlying profibrotic context [176]. In reparative fibrosis that follows myocardial infarction, the blockade of fibroblasts may have discordant effects at the periphery versus the center of the scar. Therapeutic approaches designed to block cardiac fibrosis should thus focus on preventing excessive ECM deposition at the periphery of the scar and should not affect the replacement of necrotic cardiomyocytes within the scar core. The most adequate moment for blocking the different profibrotic pathways remains another pending issue at this point. Blocking mediators at the wrong time could alter cellular responses that are critical for tissue repair. In reparative fibrosis that occurs after massive acute injury (e.g., after acute myocardial infarction), the beneficial effects of fibrotic tissue clearly outweigh its harmful effects. In such settings, early antifibrotic interventions could negatively affect the healing process and promote rupture of the heart wall, whereas delayed fibrosis inhibition may be ineffective if the fibrotic process is no longer reversible.

Numerous strategies aiming to prevent, block, and even reverse cardiac fibrosis have been extremely promising in animal models. However, their evaluation in human patients delays or, if they were tested in clinical settings, the results were rather disappointing. Interspecies discordances obviously mandate caution when trying to extrapolate data from animal studies to human medicine and can contribute to the discordant results obtained with different antifibrotic agents. Other factors may play, however, even greater roles. Drug doses used in animal studies are often much higher than those suitable for clinical use, and, with very few exceptions, currently used animal models have limited ability to adequately replicate human CVDs. Whereas in humans, CVDs are most often diseases of elderly individuals, with numerous concomitant cardiac and non-cardiac conditions, treated with different medications, including with a wide variety of cardioactive drugs, and in whom treatment adherence is often questionable, most animal data arise from young, healthy animals, fully compliant to all forms of therapy and who have no concomitant diseases and no concomitant therapy [177]. Using more clinically relevant animal CVDs models would certainly increase the translational value of data obtained in animal models. Meanwhile, clinical trials on innovative strategies have either been performed on a small number of patients or the follow-up period was much too short, considering the important interspecies differences regarding the time needed for the development of fibrosis, which seems to be much longer in humans [177]. Signaling pathways, profibrotic mechanisms, and even the type of fibrosis that develops have also been shown to vary greatly depending on the underling fibrose-promoting condition, suggesting that although most cardiac diseases involve a certain degree of fibrosis, a ‘one size fits all’ approach is unlikely to provide the solution in cardiac antifibrotic therapy. To date, with the exception of biomaterial-based approaches, which have been largely studied in post-myocardial infarction fibrosis, antifibrotic strategies have rarely been studied targeted on a specific type of fibrosis. Thorough understanding of the pathophysiological mechanisms underlying each type of myocardial fibrosis could provide the key for safe and efficient, targeted antifibrotic therapy.

Data from clinical trials confirmed the safety of stem cells from different tissue sources, using different delivery routes, but their exact clinical benefit remains to be established. Large phase III clinical trials are in progress, and their results will be essential to determine the role of this novel, non-pharmacological approach. To fully understand the potential role of stem cell therapy in cardiac fibrosis, the mechanisms by which this therapy exerts its effects will also need to be clarified. Inhibition of the RAAS using anti-Ang II vaccines, administration of Ang (1–7), and ACE2 overexpression recently emerged as a promising new tool for myocardial fibrosis management in animal models and even in small clinical trials. Antifibrotic drugs used in different other settings would also be worth consideration. Pirfenidone, nintedanib, tranilast, bosentan, macitentan, ambrisentan, and thalidomide are drugs with excellent results in pulmonary fibrosis, and only a minority of them has been evaluated so far in CVDs. Hydralazine and ivabradine, already widely used in patients with CVDs, were shown to significantly attenuate renal fibrosis, but very few studies have assessed their cardiac antifibrotic effect (Table 2). Sildenafil was reported to exert antifibrotic effects not only in the genitals but also in the lungs and skin. A similar effect on cardiac fibrosis could thus contribute to the improvement in ventricular function associated with sildenafil usage. Bioengineering and cell transplant therapy have also demonstrated major potential in indirectly blocking fibrosis by stimulating myocardial regeneration/repair. Direct cell reprogramming and molecular targets, such as epigenetic modifiers and miRs, have also been proposed as novel promising pharmacological tools to prevent the development of cardiac scar tissue. Targeting cardiac fibrosis is still associated, however, with a number of major limitations, and the mechanisms that lead to excessive ECM formation remain incompletely understood. In the absence of myocardial regeneration, the degradation of large areas of fibrosis could result in catastrophic consequences. Future studies will need to fully elucidate the mechanisms involved in cardiac fibrosis, to identify safe and effective methods to counteract this harmful process, and to establish the most appropriate time to intervene.

## 6. Conclusions

Cardiac fibrosis is currently acknowledged as a central element in the vast majority of CVDs. Due to the complexity of the signaling pathways, to its dual, protective and deleterious impact, and to the numerous cell types involved in the fibrotic process, safe and effective therapies for cardiac fibrosis inhibition and/or reversal remain difficult to develop. Continuous research in this area will have to fully elucidate the mechanisms involved in cardiac fibrosis, to identify safe and effective antifibrotic methods, and to establish the most appropriate time to intervene.

## Figures and Tables

**Table 1:** Advantages and disadvantages of different techniques used in the evaluation and quantification of cardiac fibrosis.

Technology	Advantages	Disadvantages
Echocardiography	- favorable safety profile - non-invasive - acceptable to most patients - low cost - portable	- does not allow direct identification and quantification of fibrosis type and extent - cannot be used to measure and monitor the degree and progression of myocardial fibrosis - poor reproducibility - dependent on acoustic windows - affected by operator’s skills
Cardiac magnetic resonance	- can identify macroscopic fibrosis - can identify different patterns of fibrosis - acceptable to patients - non-invasive	- potential artifacts in uncooperative patients and in the presence of tachyarrhythmias - contraindicated in patients with magnetic resonance-incompatible implants - high cost
Endomyocardial biopsy	- allows direct microscopic assessment of myocardial components and fibrotic changes	- risk of major complications - sampling error in cases of localized fibrosis - unreliable in detecting replacement fibrosis

**Table 2:** Clinical and experimental studies of drugs studied for their antifibrotic effects.

Therapeutic Class	Drug	Study Type	Species	Duration	Underlying CVD	Results	References
RAAS inhibitors	Spironolactone (12.5–50.0 mg/day)	Placebo-controlled randomized trial	Human	6 months	HFrEF	Reduced PINP/PIIINP	[77]
	Lisinopril (5–20 mg/day)	Double-blind randomized trial	Human	6 months	Hypertensive heart disease	Reduced CVF and improved diastolic function	[78]
	Enalapril (5 mg/day)	Double-blind, randomized controlled clinical trial	Human	6 months	HFpEF-ESRF	Reduced PICP	[79]
	Losartan (50 mg/day)	Double-blind, randomized controlled clinical trial	Human	6 months	HFpEF-ESRF	Reduced CVF and improved diastolic function in severe fibrosis	[79]
Angiotensin receptor neprilysin inhibitor	Sacubitril-valsartan (200mg bid)	Double-blind, randomized controlled clinical trial	Human	9 months	HFpEF	No significant change in PIIINP/MMP2	[80]
Statins	Atorvastatin (40 mg/day)	Randomized open label study	Human	6 months	HFrEF	Reduction in PIIINP levels	[81]
	Rosuvastatin (40 mg/day)	Double-blind, randomized, placebo-controlled study	Human	6 months	HFrEF	No significant change in PINP/PIIINP	[82]
Pyridones	Pirfenidone	Double-blind, randomized, placebo-controlled study	Human	52 weeks	HFpEF	Ongoing	[83]
Mast cell degranulation inhibitor	Tranilast (400 mg/kg/day)	Experimental	Rat	12 weeks	2K1C renovascular hypertension	Decreased fibrotic area to total left ventricular area ratio	[84]
Endothelin receptor blocker	Bosentan (100 mg/kg/day)	Experimental	Rat	4 weeks	Myocardial hypertrophy	Decreased histological interstitial and perivascular fibrosis	[85]
Pacemaker current inhibitor	Ivabradine (5 mg bid)	Double-blind, randomized, placebo-controlled study	Human	8 months	HFrEF	Reversed LV volumes and increased LVEF	[86]
Phosphodiesterase type 5 inhibitors	Sildenafil (100 mg/day)	Double-blind, randomized, placebo-controlled study	Human	3 months	Type 2 diabetes	Improved LV contraction parameters and reduced TGF-*β* and MCP-1	[87]
*Beta*-blocker	Propranolol (40 mg/kg/day)	Preclinical	Rat	10 weeks	Left ventricular pressure overload, hypertrophy	No significant reduction in interstitial fibrosis	[88]
Calcium channel blockers	Mibefradil (10 mg/kg/d ay)	Preclinical	Rat	6 weeks	Myocardial infarction	Decreased infarct size and perivascular fibrosis	[89]

2K1C—two-kidney, one-clip; CVD—cardiovascular disease; CVF—collagen volume fraction; ESRF—end-stage renal disease; HFpEF—heart failure with preserved ejection fraction; HFrEF—heart failure with reduced ejection fraction; LV—left ventricle; LVEF—left ventricular ejection fraction; MCP-1—monocyte chemoattractant protein-1; MMP-2—matrix metalloproteinase-2; PICP—carboxy-terminal propeptide of procollagen type I; PINP—amino-terminal propeptide of procollagen type I; PIIINP—amino-terminal propeptide of procollagen type III; RAAS—renin–angiotensin–aldosterone system; TGF-*β*—transforming growth factor-*beta.*

**Table 3:** Novel targets for cardiac fibrosis prevention and therapy.

Therapeutic Target	Strategy
Cell transplantation	Direct remuscularization Stimulation of endogenous cardiovascular progenitor cells
TGF-*β* signaling	Suppression of TGF-*β*1 TGF*β*RII plasmid transfection ALK5 inhibition TGF*β*RII inhibition
Biomaterials	Hydrogel (alginate, polyester-VEGF, decellularized ECM, gelatin-HGF) Patch (alginate-neonatal rat cardiomyocytes, decellularized ECM) Glue (fibrin-fibroblast growth factor) Scaffold (fibrin–endothelial cells–smooth muscle cells)
Direct reprogramming	GMT (retrovirus/lentivirus) GMHT (retrovirus) miRNAs (miR-1, miR-133, miR-208, miR-499) Chemical/small molecule cocktails

ALK5—transforming growth factor-*beta* 1 type I receptor kinase; ECM—extracellular matrix; GMHT—Gata4/Mef2c/Hand2/Tbx5; GMT—Gata4/Mef2c/Tbx5; HGF—hepatocyte growth factor; TGF-*β*1—transforming growth factor-*beta* 1; TGF*β*RII—transforming growth factor-*beta* receptor II; VEGF—vascular endothelial growth factor.

## Data Availability

Not applicable.
